# The Behavioral Regulation in Exercise Questionnaire (BREQ-3) Portuguese-Version: Evidence of Reliability, Validity and Invariance Across Gender

**DOI:** 10.3389/fpsyg.2018.01940

**Published:** 2018-10-11

**Authors:** Luis Cid, Diogo Monteiro, Diogo Teixeira, Pedro Teques, Susana Alves, João Moutão, Marlene Silva, António Palmeira

**Affiliations:** ^1^Sport Sciences School of Rio Maior (ESDRM-IPSantarém), Rio Maior, Portugal; ^2^Research Centre in Sports Sciences, Health Sciences and Human Development (CIDESD), Vila Real, Portugal; ^3^Faculty of Physical Education and Sport (ULHT), Lisbon, Portugal; ^4^Research Centre, N2i, Polytechnic Institute of Maia (IPMaia), Maia, Portugal; ^5^Interdisciplinary Center for the Study of Human Performance (CIPER), Universidade de Lisboa, Cruz Quebrada, Portugal; ^6^Life Quality Research Centre, Centro de Investigação em Qualidade de Vida, Santarém, Portugal

**Keywords:** motivation, self-determination, self-regulation, exercise, multi-group analysis

## Abstract

This study has as prime objective to analyze the psychometric properties of the Behavioral Regulation Exercise Questionnaire (BREQ-3) in a sample of Portuguese exercisers and invariance across gender. Two independent samples (448 calibration; 374 validation), aged between 16 and 78 years (*M* = 40.29; *SD* = 16.24), of both gender, (495 female; 327 male) were enrolled in this study. The results show that the original model (six factors; 24 items) did not fit to the data in a satisfactory way (χ^2^ = 977.49; df = 237; B-S *p* < 0.001; SRMR = 0.07; NNFI = 0.80; CFI = 0.83; RMSEA = 0.08; 90% CI = 0.08–0.09). After removing six items (one for each factor), the model (six factors; 18 items) adjustment improved in a satisfactory way in both samples: calibration (χ^2^ = 331.86; df = 120; B-S *p* < 0.001; SRMR = 0.06; NNFI = 0.91; CFI = 0.93; RMSEA = 0.06; 90% CI 0.06–0.07) and validation (χ^2^ = 254.08; df = 120; B-S *p* < 0.001; SRMR = 0.04; NNFI = 0.93; CFI = 0.95; RMSEA = 0.06; 90% CI = 0.05–0.06). Results also showed model invariance across gender (ΔCFI ≤ 0.01). The Portuguese version of BREQ-3 (six factors; 18 items) is a valid and reliable measurement instrument to measure behavior regulation underlying self-determination theory in the exercise domain. However, the evidence also indicated that additional studies are needed to address the fragilities of the original model (six factors; 24 items).

## Introduction

Several mainstream theories have been used to study motivational processes in different contexts. Self-Determination Theory (SDT: Deci and Ryan, 2000) has been widely used to study participant’s motivation to exercise (Markland and Tobin, 2010; Ng et al., 2012). The authors of SDT postulate that two types of motivation influence personal behavior: the intrinsic motivation (doing a task for the inherent pleasure) and extrinsic motivation (doing an activity for instrumental reasons, obtaining separable outcomes or to avoid disapproval) (Sebire et al., 2009; Ryan and Deci, 2017). The extrinsically motivated behaviors are expressed in four regulations: external regulation (influenced by external contingencies), introjected regulation (performing to obtain social approval or avoiding internal pressure), identified regulation (recognition and acceptance of the behavior) and the integrated regulation (accepting and integrating behavior in others aspects of the self) (Deci and Ryan, 2000). In SDT, these regulatory mechanisms indicate degrees of behavior internalization, reflecting the transitioning of habits and requests into endorsed values and self-regulations. This presents as particularly important in the study of exercise behavior. As this process is progressively successful, exercisers may vary between controlled (extrinsic and introjected regulations) to autonomous motivation (identified and integrated regulations) (Deci and Ryan, 2000). The latter represent well-internalized extrinsic motivation, which alongside with intrinsic motivation, have been highlighted as important factors in continuous exercise adherence (Ryan and Deci, 2017).

Several instruments have been developed to measure these essential variables in different domains. The Behavioural Regulation in Exercise Questionnaire (BREQ) proposed by Mullan et al. (1997) was a first attempt to develop an instrument capable of tapping behavioral regulation according to SDT in the exercise domain. Limitations in accessing the full spectrum of behavioral regulations, particularly in the amotivation factor, led to the inclusion of four new items to surpass this limitation (Markland and Tobin, 2004). This new measure was called BREQ-2 and has become one of the most widely use instruments in exercise motivation studies. This questionnaire is composed of a 19-item scale with five factors (amotivation, external, introjected, identified and intrinsic motivation) and have been validated with a sample of 201 exercisers. Both factor structure and internal consistency presented reasonable scores.

In Portugal, BREQ-2 was translated and validated in a sample of 703 Portuguese exercisers, presenting good model fit and internal consistency (Palmeira et al., 2007), maintaining itself as one of the main instruments used in the analysis of behavioral regulations in this particular domain. A few years later, Cid et al. (2012) tested psychometric properties and also a hierarchical model that includes two second-order factors that represent an index of autonomous and controlled motivation in a sample of 550 Portuguese gym and health club exercisers. The results supported the use of Portuguese BREQ-2 in exercise for the evaluation of behavioral regulation underlying SDT, as well as for the assessment of autonomous (intrinsic and identified) and controlled (external and introjected) motivation.

However, one of the main issues regarding BREQ-2 was the inability to access one of the SDT proposed behavioral regulations (integrated regulation). For this matter, Wilson et al. (2006) suggested the inclusion of the integrate subscale in BREQ-2, allowing the complete analysis of the behavioral regulations proposed by SDT framework. The integrated subscale (reflecting personal endorsed values, goals and needs) is the most autonomous form of extrinsic motivation, reflecting congruence between behavior regulation and the self (Deci and Ryan, 2000; Wilson et al., 2006). The implications of the analysis of this regulation seems undisputable, as it allows a better and refined understating of the extrinsically motivated exercisers (particularly in the gap between accepting the behavior and obtaining a separable and pleasurable outcome), and the ability to capture SDT’s motivational continuum in exercise.

Therefore, the analysis of the feasibility of extending the BREQ-2 and its application in the Portuguese exercise domain determines its two main objectives: (1) to validate the Behavioral Regulation Exercise Questionnaire (BREQ-3) in a Portuguese sample of gym exercisers and (2) to analyze model invariance across gender.

## Materials and Methods

### Participants

Two independent samples of gym exercisers were enrolled in this study from several types of fitness activities provided in gym, such as: weight training, group activities (e.g., indoor cycling, aerobic, step, pump, combat), cardio-fitness activities (i.e., combined strength training and aerobic activities), and water activities. With an average age of 40.29 (*SD* = 16.24) years old in both samples, the years of practice ranged between 0.3 and 25 years (*M* = 7.34; *SD* = 7.25), with an average of 2.8 sessions per week (*SD* = 1.03) and exercise sessions ranged between 60 and 180 min per session. The first sample consisted of 448 subjects and reflected the calibration sample; the second sample consisted of 374 subjects and reflected the validation sample, to prove the robustness of the measurement instrument in a different sample with the same characteristics. The samples are characterized as follows: (a) calibration sample: this sample was composed of 448 exercisers enrolled in several activities (266 female; 182 male) aged between 16 and 78 years old (*M* = 39.96; *SD* = 16.25); (b) validation sample: this sample was composed of 374 exercisers enrolled in several activities (229 female; 145 male), aged between 17 and 77 years old (*M* = 40.51; *SD* = 16.07); (c) male sample: this sample consisted of 327 exercisers of different activities, aged between 16 and 78 years (*M* = 38.60; *SD* = 15.92); (d) female sample: this sample consisted of 495 exercisers of different activities, aged between 16 and 77 years (*M* = 41.27 and 16.74, respectively). Before data collection, Ethical approval was obtained from the committee of the Research Center in Sports Sciences, Health Sciences and Human Development (CIDESD), unit that is registered in the Portuguese National Science Foundation (FCT) under the reference UID/DTP/04045/2013.

### Measures

The Behavioral Regulation Exercise Scale (BREQ-2: Markland and Tobin, 2004). For this study, we used the Portuguese version of BREQ-2, translated and preliminarily validated by Palmeira et al. (2007) and validated by Cid et al. (2012), to include an integrated regulation scale (Wilson et al., 2006). This questionnaire (BREQ-3) consisted of 24 items^1^ with a five-point Likert scale, which varied between 1 (“Strongly Disagree”) and 4 (“Strongly Agree”). The items were grouped posteriorly into six factors (with four items each), which reflected the motivational continuum of SDT (Deci and Ryan, 2000).

### Procedures: Data Collection

Permission to collect information at gyms was given by the administrators. The researchers approached randomly selected prospective participants in the reception area before exercise sessions and at the end of the day when most individuals frequented the gyms. All participants provided signed informed consent. Confidentiality were granted and assured, clarifying that the information would not be released to third parties. After a short explanation of the study general objective, the assessment instrument was applied separately to each participant, which took approximately 15 min.

### Procedures: Translation of the Integrated Regulation Subscale

For the translation and adaptation of the four item integrated subscale (Wilson et al., 2006) from the original language (English) to the Portuguese language, we adopted methodological procedures suggested by Vallerand (1989). However, instead the translation/back translation technique proposed by Vallerand (1989) was used the committee approach methodology (Brislin, 1980), developed in five stages.

### Data Analysis

The analysis was performed using a Confirmatory Factor Analysis (CFA) according to the recommendations of several authors (Marsh et al., 2004; Byrne, 2010; Hair et al., 2014), using as method of estimation the maximum likelihood (MLE) through chi-square test (χ^2^), degrees of freedom (*df*) and significance levels (*p*), and also the following goodness-of-fit indices: standardized root mean square residual (SRMR), comparative fit index (CFI), non-normed fit index (NNFI), root mean square error of approximation (RMSEA) and respective confidence interval (RMSEA 90% CI). In the present study, and for the aforementioned indices, the following cut-off values were adopted: SRMR ≤ 0.08, CFI and NNFI ≥ 0.90, and RMSEA ≤ 0.08 (Marsh et al., 2004; Byrne, 2010; Hair et al., 2014). Analyses were carried out using AMOS 20.0 software.

Convergent validity was analyzed via the calculation of the average variance extracted (AVE), considering values of AVE ≥ 0.50. Discriminant validity was also analyzed and was establish when the AVE for each construct exceeded the squared correlation between that construct and any other. Finally, composite reliability (CR) was analyzed and adopted CR ≥ 0.70 as a cut-off values, as suggested by Hair et al. (2014). Convergent validity was analyzed via the calculation of the AVE, considering values of AVE ≥ 0.50.

Additionally, the multi-group analysis was conducted to assess whether the measurement model structure was equivalent in different groups with different characteristics (calibration vs. validation samples and male vs. female samples). Thus, the following criteria were established for the invariance of the models: (Cheung and Rensvold, 2002; Byrne, 2010): (1) a factorial model analysis for each group individually and (2) a multi-group analysis by restricting the model parameters, considering the following types of invariance: the free parameters model (configural invariance), the fixed factorial measurement model (measurement invariance), the fixed factorial and covariance measurement model (scale-invariance) and the fixed factorial, covariance and error measurement model (residual invariance).

According to Marsh (1993), when analyzing models with this procedure, the measurement invariance is considered a minimal criterion for the invariance of the model, and the residual invariance (last criterion) is not suggestive of a lack of model invariance. Some authors even considered that the analysis of this criterion was infrequent due to it being too restrictive (Byrne, 2010). As suggested by Cheung and Rensvold (2002), the difference in values between the unrestricted and the restricted model (i.e., free parameters vs. fixed parameters) should be ΔCFI ≤ 0.01.

## Results

A preliminary analysis of the data revealed 10 missing value cases. These participants were removed prior to conducting the analysis, as advocated by several authors (Hair et al., 2014). As presented in **Table 1**, individuals that used all answer levels (from 0 to 4) had higher means associated with items related to identified and integrated regulation and intrinsic motivation subscales. These answers also depicted a non-normal univariate distribution of the data, which presented a bias to the left, and could be explained by the tendency for the individuals to use the highest levels of an answer (i.e., three and four) in this kind of questionnaire.

**Table 1:** Descriptive analysis of the answers to the items on the BREQ-3 for the Portuguese sample.

	*M*	*SD*	Skewness	*z*-value	Kurtosis	*z*-value
Item 1 (AM)	0.46	1.05	2.31	0.19	4.25	0.72
Item 2 (EX)	0.49	0.95	2.07	0.17	3.74	0.63
Item 3 (IJ)	1.65	1.36	0.28	0.02	−1.02	−0.17
Item 4 (ID)	3.44	0.92	−1.74	−0.14	2.54	0.43
Item 5 (IG)	2.81	1.22	−0.79	−0.06	−0.28	−0.04
Item 6 (IM)	2.74	1.26	−0.76	−0.06	−0.40	−0.06
Item 7 (AM)	0.33	0.85	2.83	0.24	7.50	1.27
Item 8 (EX)	0.49	0.87	2.03	0.17	3.99	0.67
Item 9 (IJ)	0.78	1.07	1.37	0.11	1.21	0.20
Item 10 (ID)	3.23	1.07	−1.31	−0.11	0.89	0.15
Item 11 (IG)	2.70	1.23	−0.64	−0.05	−0.51	−0.08
Item 12 (IM)	3.38	0.92	3.17	0.26	3.36	0.57
Item 13 (AM)	0.29	0.79	2.90	0.24	9.973	1.69
Item 14 (EX)	0.30	0.73	0.34	0.02	9.05	1.53
Item 15 (IJ)	1.60	1.44	−0.97	−0.08	−1.22	−0.20
Item 16 (ID)	2.93	1.24	−0.62	−0.05	−0.09	−0.01
Item 17 (IG)	2.68	1.25	−1.61	−0.13	−0.60	−0.10
Item 18 (IM)	3.24	1.07	4.37	0.37	2.08	0.35
Item 19 (AM)	0.17	0.62	2.80	0.23	20.43	3.47
Item 20 (EX)	0.33	0.77	0.32	0.02	8.15	1.38
Item 21 (IJ)	1.63	1.37	−0.07	−0.005	−1.10	−0.18
Item 22 (ID)	2.09	1.34	−0.79	−0.06	−1.12	−0.19
Item 23 (IG)	2.88	1.08	−1.98	−0.16	0.07	0.01
Item 24 (IM)	3.55	0.78	3.17	0.26	4.53	0.77

*AM, amotivation; EX, external regulation; IJ, introjected regulation; ID, identified regulation; IG, integrated regulation; IM, intrinsic motivation; M, mean; SD, standard deviation.*

Moreover, Mardia’s coefficient for multivariate kurtosis exceeded expected values multivariate normality assumption (>5.0) in all samples (Byrne, 2010). As suggested in literature, Bollen-Stine bootstrap with 2000 samples was employed for subsequent analysis (Nevitt and Hancock, 2001).

**FIGURE 1:**
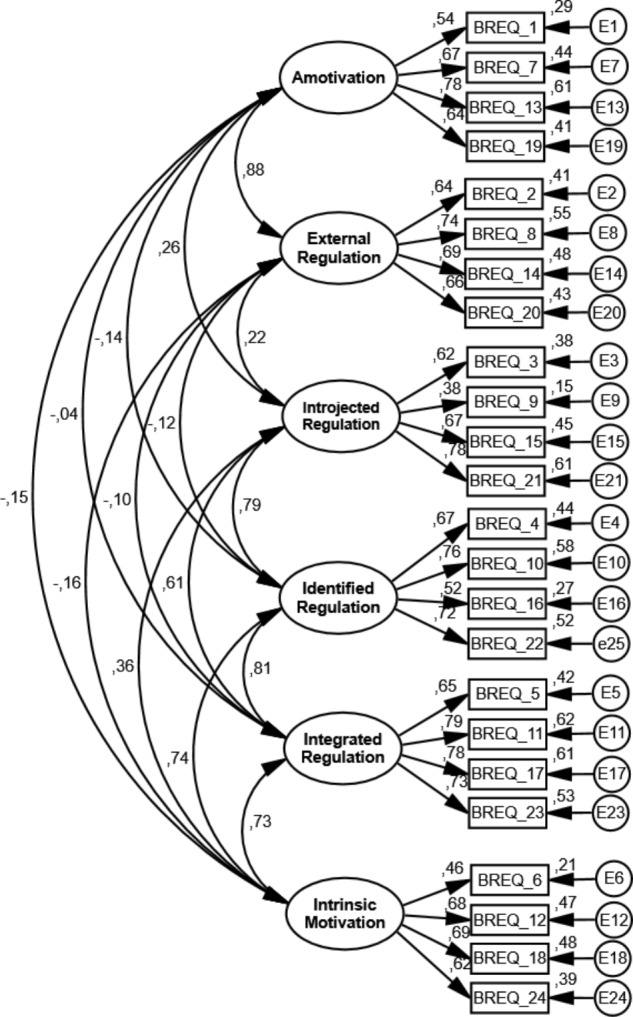
Standardized individual parameters (covariance factors, factorial weights and measurement errors), all of which were significant in the measurement model (BREQ-3 – six factors and 24 items) for the calibration sample initial model.

As seen in **Table 2**, the initial model (six factors and 24 items – **Figure 1**) did not fit to the data. Potential issues were sought through the analysis of the residual values between the items and the modification indices, obtaining a better adjusted model with six items removal (one for each factor), after which the model’s adjustment indices improved slightly (**Table 2**). After this procedure, the measurement model fit to the data, being in agreement with the cut-off values suggested in the methodology for each of the analyzed samples.

**Table 2:** Goodness-of-fit indices of BREQ models (including others existing versions).

	χ^2^	*df*	χ^2^*/df*	SRMR	NNFI	CFI	RMSEA	RMSEA 90% CI
English version (BREQ-3)^a^	253.82^∗^	142	1.79	0^−^	–	0.93	0.09	0.07–0.09
Brazilian version (BREQ-3)^b^	406.35^∗^	215	1.89	0^−^	–	0.93	0.07	0.06–0.07
Spanish version (BREQ-3)^c^	689.13^∗^	215	3.21	0.06	–	0.91	0.06	–
Portuguese version (BREQ-2)^d^	221.70^∗^	125	1.77	0.06	0.90	0.92	0.04	0.03–0.05
Present study initial model CS	977.49^∗∗^	237	4.12	0.07	0.80	0.83	0.08	0.08–0.09
Present study final model CS	331.86^∗∗^	120	2.77	0.06	0.91	0.93	0.06	0.06–0.07
Present study final model VS	254.08^∗∗^	120	2.12	0.04	0.93	0.95	0.06	0.05–0.06
Present study male model	282.18^∗∗^	120	2.35	0.06	0.91	0.93	0.07	0.06–0.08
Present study female model	335.14^∗∗^	120	2.79	0.04	0.92	0.94	0.06	0.05–0.07

*χ^2^, chi-squared; df, degrees of freedom; χ^2^/df, normative chi-square; SRMR, standardized root mean square residual; NNFI, non-normed fit index; CFI, comparative fit index; RMSEA, root mean squared error of approximation; 90% CI, confidence interval of RMSEA; ^a^Wilson et al. (2006); ^b^Guedes and Sofiati (2015); ^c^González-Cutre et al. (2010); ^d^Cid et al. (2012); initial model (six factors/24 items); final model (six factors, 18 items); CS, calibration sample; VS, validation; ^∗^ = p < 0.001, ^∗∗^ = Bollen-Stine Bootstrap (B-S) p < 0.001.*

**FIGURE 2:**
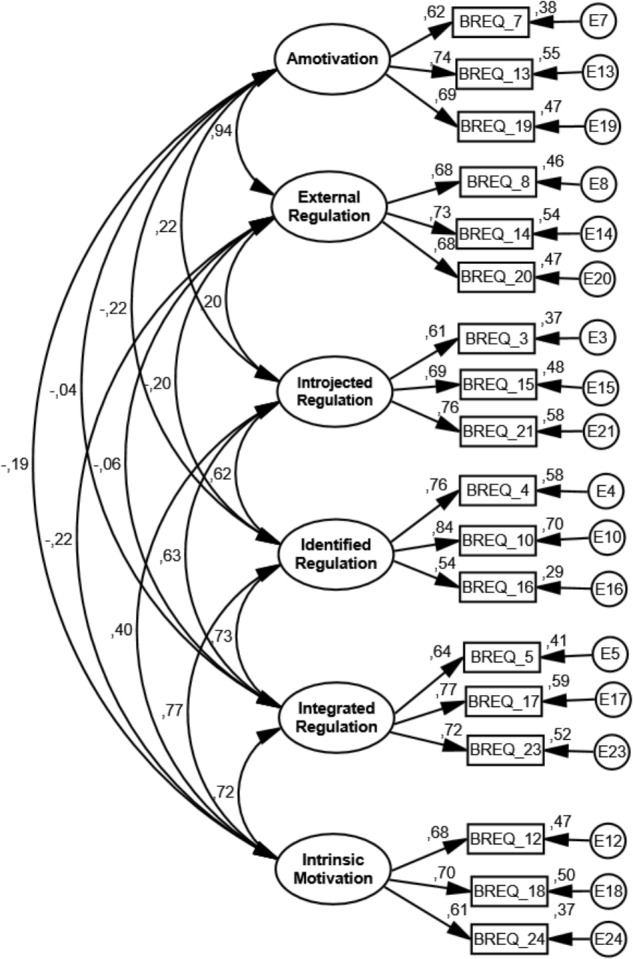
Standardized individual parameters (covariance factors, factorial weights and measurement errors), all of which were significant in the measurement model (BREQ-3 – six factors and 18 items) for the calibration sample final model.

**FIGURE 3:**
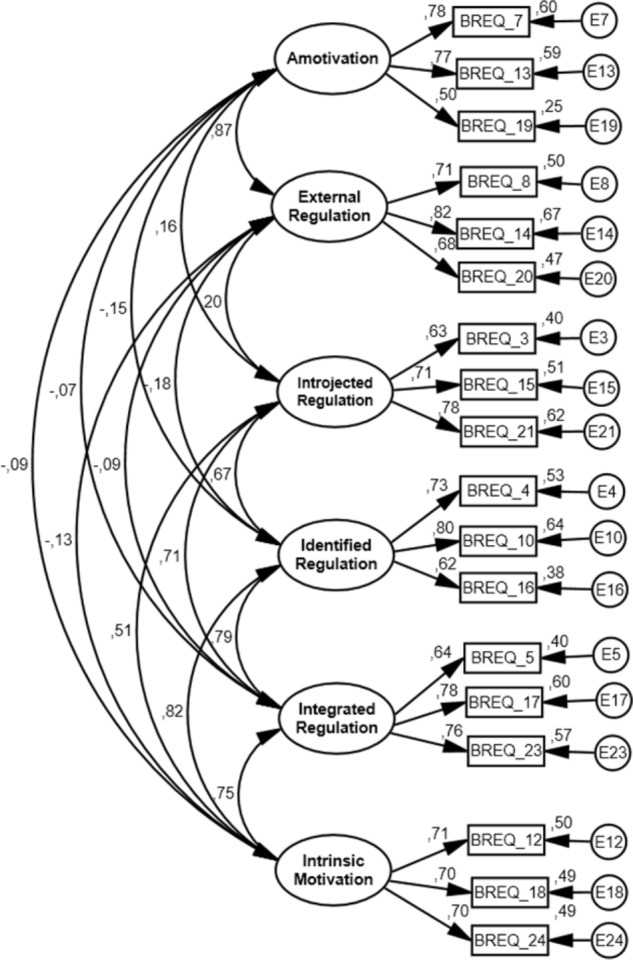
Standardized individual parameters (covariance factors, factorial weights and measurement errors), all of which were significant in the measurement model (BREQ-3 – six factors and 18 items) for the validation sample final model.

According to the results presented in **Figure 2** (calibration sample final model) and **Figure 3** (validation sample final model), we verify in the first place that the correlation patterns between the different types of motivation evidence a simplex structure. In other words, the regulation types closer through the continuum are positively correlated among them, and those that are farther correlate less positively or negatively (Ryan and Connell, 1989; Howard et al., 2017). Relative to the adjustment of the model’s individual parameters, factorial validity was present (all items had a factorial weight on the respective factor and all statistically significant; *p* < 0.05). For the calibration sample final model (six factors and 18 items – **Figure 2**), the factorial weights varied from 0.62 to 0.74 (amotivation); 0.68 to 0.73 (external regulation) 0.61 to 0.76 (introjected regulation); 0.54 to 0.84 (identified regulation); 0.64 to 0.77 (integrated regulation); and 0.61 to 0.70 (intrinsic motivation). For the validation sample final model (six factors and 18 items – **Figure 3**), the factorial weights varied from 0.50 to 0.78 (amotivation); 0.68 to 0.82 (external regulation); 0.63 to 0.78 (introjected regulation); 0.62 to 0.78 (identified regulation); 0.64 to 0.78 (integrated regulation); and 0.70 to 0.71 (intrinsic motivation). Furthermore, more than 25% of the variance of the latent factor were explained by all items, a value commonly accepted (Hair et al., 2014).

According to **Table 3**, all the factors underlying the measurement model presented an adjusted composite reliability (≥0.70) in both calibration and validation samples. Regarding the convergent validity, minor issues were found in the calibration (amotivation, external and introjected regulations and intrinsic motivation factors) and validation samples (i.e., amotivation and intrinsic motivation factors), because the values of AVE were inferior to the value adopted in the methodology (Hair et al., 2014). In respect to the discriminant validity, issues between AM-EX, ID-IG, and ID-IM for the calibration sample and between AM-EX, ID-INTG, ID-MI, and IG-MI for the validation sample were found, because the square of the factor’s correlation between these factors were higher than the AVE (Hair et al., 2014).

**Table 3:** Internal reliability, convergent and discriminant validity, and average variance extracted of calibration and validation samples.

	CR	AVE	AM	EX	IJ	ID	IG	IM
Calibration sample								
Amotivation (AM)	0.72	0.47	1	0.89	0.04	0.05	0.00	0.04
External (EX)	0.74	0.49	–	1	0.04	0.04	0.00	0.04
Introjected (IJ)	0.73	0.48	–	–	1	0.38	0.40	0.16
Identified (ID)	0.77	0.52	–	–	–	1	0.53	0.58
Integrated (IG)	0.75	0.51	–	–	–	–	1	0.51
Intrinsic (IM)	0.70	0.45	–	–	–	–	–	1
**Validation sample**								
Amotivation (AM)	0.73	0.48	1	0.76	0.03	0.02	0.01	0.01
External (EX)	0.77	0.55	–	1	0.04	0.03	0.04	0.02
Introjected (IJ)	0.75	0.51	–	–	1	0.44	0.50	0.26
Identified (ID)	0.76	0.52	–	–	–	1	0.63	0.68
Integrated (IG)	0.77	0.52	–	–	–	–	1	0.56
Intrinsic (IM)	0.75	0.49	–	–	–	–	–	1

*Correlation among factors (in diagonal) represents the squared values (r^2^); CR, composite reliability; AVE, average variance extracted; AM, amotivation; EX, external regulation; IJ, introjected regulation; ID, identified regulation; IG, integrated regulation; IM, intrinsic motivation.*

The data from **Table 4** indicates that the model was invariant across samples (showing evidences of cross-validation) and gender (the final model is equivalent across male and female samples). The results also indicate the following: the same number of factors was present in all groups, with each factor associated with the same group of items (measurement invariance); BREQ-3 factors had the same meaning for both groups (metric invariance); the comparison of the latent and observable means was valid among the groups (scale invariance); and comparison between observable items is assured (residual invariance).

**Table 4:** Goodness-of-fit indices for invariance of the BREQ-3 across gender and across calibration and validation samples.

	χ^2^	df	χ^2^/df	Δχ^2^	Δ*df*	*p*	CFI	ΔCFI
**Male sample - female sample**								
Configural Invariance	702.72	240	2.93	–	–	–	0.917	–
Measurement Invariance	721.71	252	2.86	18.98	12	0.089	0.915	0.002
Scale Invariance	769.29	273	2.82	66.56	33	0.001	0.911	0.006
Residual Invariance	842.10	291	2.89	139.38	51	0.001	0.901	0.016
**Calibration sample– validation sample**								
Configural Invariance	585.94	240	2.44	–	–	–	0.936	–
Measurement Invariance	613.17	252	2.43	27.23	12	0.005	0.933	0.003
Scale Invariance	625.16	273	2.29	39.22	33	0.007	0.935	0.006
Residual Invariance	710.30	291	2.44	124.36	51	0.022	0.923	0.013

*χ^2^, chi-squared; df, degrees of freedom; χ^2^/df, normative chi-square; Δχ^2^, differences in the value of chi-squared; Δdf, differences in the degrees of freedom; CFI, comparative fit index; ΔCFI, differences in the value of the comparative fit index.*

## Discussion

Taking into account the study objective, the validation of the Portuguese version of BREQ-3 in a sample of exercisers, as well as evidence of criteria of cross-validity between samples and invariance between gender, increases the scientific evidence contributing to what (Deci and Ryan, 2008) designated as the “development of knowledge about the universality of the variables underlying the theory of self-determination,” that in this case, refers to the regulation of motivation in the exercise domain.

In the descriptive analysis, the results show that the participants tend to value the items of the questionnaire, which in fact seems to be demonstrated by the moderate and high averages in all of them; thus, evidencing the theoretical importance underlying the motivational continuum of the SDT. These results are in line with BREQ validations in other languages (Markland and Tobin, 2004; González-Cutre et al., 2010; Moustaka et al., 2010; Cid et al., 2012; Guedes and Sofiati, 2015; Liu et al., 2015).

Regarding the psychometric properties of BREQ-3 for a sample of Portuguese exercisers, the results showed that the initially hypothesized model (six factors and 24 items) did not fit the data according to the values adopted in the methodology (Marsh et al., 2004; Byrne, 2010; Hair et al., 2014). Bearing this in mind, individual parameters were analyzed, based on residual values and modification indices of the Lagrange test, and items 1 (amotivation – “*I don’t see why I should have to exercise*”); 2 (external regulation – “*I exercise because other people say I should*”); 6 (intrinsic motivation – “*I exercise because it’s fun*”); 9 (introjected regulation – “*I feel ashamed when I miss an exercise session*”); 11 (integrated regulation – “*I consider exercise to be part of my identity*”) and 22^2^ (identified regulation – “*I value exercise and I get restless if I don’t exercise regularly*”) were removed due to: (1) standardized residual matrix showed high residual values between mentioned items and other types of behavior regulations items, and (2) modification indices found cross-loadings between mentioned items and other factors.

Comparing the results of the present study with the results of other BREQ3 versions, we verified that there is some contradiction regarding the final structure of the measurement model. In the Portuguese version of BREQ3, the model only adjusted to the data after the elimination of some items, which did not happen in the Spanish and Brazilian versions. The Spanish version of BREQ3 (González-Cutre et al., 2010) used a sample that includes practitioners from different exercise contexts, being slightly different from the one used in the present study, which may explain some differences found in the initial model adjustment. The Brazilian version of BREQ3 (Guedes and Sofiati, 2015), whose content of the items in the questionnaire is very close to those of the Portuguese version, used a sample very similar to the one used in the present study, and the original model fit the data. However, it is interesting to note that the Brazilian version of the BREQ2 (Klaine et al., 2015), also validated in a sample of gym exercisers, only adjusted to the data after the elimination of two items (one of intrinsic motivation and one of identified regulation), suggesting inconsistencies in some items. Similar results were found in a recent study carried out with a sample of patients diagnosed with schizophrenia (Costa et al., 2017), who identified problems in the Portuguese version of BREQ-3 structure (i.e., cross-loading between some items of controlled and autonomous motivation, particularly, in introjected and identified regulations), which highlights the need to further develop studies than can improve and refine the use of this scale.

In sum, the items mentioned above are the ones that showed higher fragilities, which led to their elimination. After this procedure, the final model (six factors and 18 items) fitted the data, in all samples according to the values adopted (Marsh et al., 2004; Byrne, 2010; Hair et al., 2014).

Taking into account previous studies performed with the Portuguese version of BREQ-2, we can verify that item 1 (amotivation) and item 6 (intrinsic motivation) had a lower factorial weight in the study done by Cid et al. (2012), and item 9 (introjected regulation) had a lower factorial weight in the preliminary study (Palmeira et al., 2007).

However, the greatest weaknesses were found with item 22 (identified regulation) (corresponding to item 17 of BREQ-2). This item proved to be more inconsistent (because it was not associated with the factor for which it was supposed to be associated), either in the original version (Markland and Tobin, 2004) or in the Portuguese version (Cid et al., 2012), as well as in the Spanish version (Moreno-Murcia et al., 2007; González-Cutre et al., 2010), in the Greek version (Moustaka et al., 2010) and in the Chinese version (Liu et al., 2015). In fact, this fragility led to the elimination of this item in the studies of González-Cutre et al. (2010) and Cid et al. (2012) suggesting that future work by other authors should readjust the semantic value of the item and test a new version of it, as was done in the present study.

In addition, given the conceptual nature of item 22 (item 17 of BREQ-2) in the validation study of the Spanish version of BREQ-3 (González-Cutre et al., 2010) the authors associated the item (with the original content) with the “introjected regulation” factor, and the model adjusted to the data. This was also the strategy used in the present study, which obtained the same result regarding this item. In fact, results tend to suggest that individuals may have understood this item as referring to introjected rather than identified motivation. Accordingly with some definitions found in literature, (Ryan and Connell, 1989; Deci and Ryan, 2000; Ryan and Deci, 2017), the description in item 22 (“*Because I get restless if I don’t exercise regularly*”) is closer to the introjected regulation definition (the individual engages in the activity due to internal pressures and to avoid feelings of guilt and/or anxiety) than to identified regulation (although not enjoying the activity itself, the individual values the activity as personally important and inherently valuable).

The results also showed that the questionnaire presents good psychometric qualities, which according to Hair et al. (2014) relates mainly to construct validity, because a set of items reflects the latent theoretical constructs expected to be measured. As far as reliability, all factors showed good internal consistency, with values of composite reliability ≥ 0.70 (Hair et al., 2014). Nevertheless, the questionnaire revealed small problems of convergent validity (values close to the cut-off value) in the amotivation factor, external regulation and introjected and intrinsic motivation factors in the calibration sample. However, the validation sample only revealed problems in the amotivation and intrinsic motivation factors, since stroke values were lower than the recommended value adopted in the methodology (AVE ≤ 0.50) (as suggested by some authors, e.g., Hair et al., 2014), although all factorial weights of this construct are equal to or greater than 0.54 (calibration sample) and 0.50 (validation sample) and all are statistically significant (*p* ≤ 0.05). These results are in line with the Brazilian (Guedes and Sofiati, 2015) and Spanish (González-Cutre et al., 2010) versions, showing similar internal consistency values (Cronbach’s alpha between 0.66 and 0.87, and between 0.68 and 0.86, in the Spanish and Brazilian versions, respectively) and minor issues in convergent validity (particularly in the introjected and identified regulations). According to Hair et al. (2014) if the factorial weights are ≤ 0.50 and statistically significant, the factors have a good convergent validity, as seen in the present study. In addition, according to Byrne (2010), when the items are significant in the respective factor, this is an indicator of the non-existence of cross-loadings, making it possible to affirm that the factors present convergent validity.

In relation to invariance across samples (cross-validation) and across gender, the best practices recommended by several authors (Byrne, 2010) regarding the re-specification of the model were followed, which recommend that if a hypothesized model does not present an adjustment to the data, the final (re-specified) model, should be tested in another sample (of the same population) to prove its validity and robustness. Thus, the final model, resulting from the analysis performed on the calibration sample was tested on another sample from the same population (validation sample). In this way, the final model was adjusted to the data according to values adopted in the method section (Marsh et al., 2004; Byrne, 2010) since it was invariant between the calibration sample and the validation sample.

On other hand, the final model also showed evidence of gender invariance, which confirms the equivalence of the model across male and female exercisers, as all criteria adopted in the methodology were met (with the exception of residual invariance). However, there seems to be no consensus on the need to evaluate residual invariance (Byrne, 2010); so the evaluation of this assumption is considered optional by the researcher, because it is too restrictive and difficult to achieve in research in social sciences, not meaning, therefore, lack of invariance (Cheung and Rensvold, 2002). These results demonstrate that the theoretical constructs underlying the measured model (motivational continuum of SDT) are perceived in the same way between male and female exercisers and comparisons can be made between them (Sass, 2011).

In conclusion, study main findings revealed that the six factors and 24 items of the Portuguese BREQ-3 measurement model did not have acceptable psychometric properties, because this model exhibited a poor fit to the data. After re-specification of the model by the elimination of six items (one item for each factor), the final model (six factors and 18 items) showed evidence of validity, reliability and invariance across gender, and can be used to measure behavior regulation underlying the SDT in the exercise domain. However, results must be contextualized to the sample in study because the evidence also indicated that additional studies are needed to confirm the psychometric properties of the model in others samples of gym exercisers, especially to address the fragilities of the original model (six factors and 24 items).

According to Vlachopoulos et al. (2013) SDT is a good example of a theory that has been developed considering cross-cultural applicability. This means that SDT constructs are universal in their importance and their effects (Deci and Ryan, 2008). This assumption has been highlighted in literature, namely in cross-cultural studies (Vlachopoulos et al., 2013; Cid et al., 2016) and in studies that analyzed the effects of a self-determined motivation using BREQ-3 (Zafeiridou et al., 2014; Sevil et al., 2016). However, future studies are encouraged to examine the universality of this measure, especially across different countries and cultures (e.g., western and eastern), as suggested by Liu et al. (2015). Without construct equivalence, a cross-cultural comparison is not recommended. Thus, researchers should further study the constructs structure and items adequacy, ensuring they have the same cultural significance (He and van de Vijver, 2012).

## Author Contributions

LC, DM, JM, and DT were enrolled in study design, data collection, and writing of the first draft manuscript. LC, DM, and PT participated in data analysis and writing of the methodology and results. DT, SA, MS, and AP participated in data collection and in final revisions of the manuscript. All authors read and approved the final version of the manuscript, and agreed with the order of presentation of the authors.

## Conflict of Interest Statement

The authors declare that the research was conducted in the absence of any commercial or financial relationships that could be construed as a potential conflict of interest. The handling Editor declared a past co-authorship with one of the authors PT.

## Footnotes

1 19 items of BREQ-2 (Markland and Tobin, 2004), four items of integrated regulation scale (Wilson et al., 2006), and one new item of introjected regulation scale to address the limitations of BREQ-2 related with item 17 pointed out in Cid et al. (2012). In BREQ-2, item 17 of the identified regulation scale (“*I get restless if I don’t exercise regularly*”) was excluded from the analysis due to an unspecified error in the original version (Markland and Tobin, 2004), and also excluded from the Portuguese version (Cid et al., 2012) because it does not fit to the identified regulation scale and shows high association with the introjected regulation scale. So, in the present study, we associated the original item 17 with the introjected regulation scale. This strategy was adopted also in the Spanish version of BREQ-3 (González-Cutre et al., 2010) and in the present study leads to development of a new item permitting us to have four items in which to factor (introjected regulation scale was the only one with 3 items): “*I value exercise and I get restless if I don’t exercise regularly.*”

2 New item of identified regulation developed to address the problems found with original item: “*I get restless if I don’t exercise regularly.*”
